# The influence of LV geometry on the occurrence of abnormal exercise tests in athletes

**DOI:** 10.1186/s12872-018-0983-1

**Published:** 2019-01-06

**Authors:** Danny A. J. P. van de Sande, Jan Hoogsteen, Pieter A. Doevendans, Hareld M. C. Kemps

**Affiliations:** 1 Department of Cardiology, Máxima Medical Center, De Run 4600, 5504 DB Veldhoven, The Netherlands; 2 Department of Cardiology, University Medical Center Utrecht, Utrecht, The Netherlands

**Keywords:** Athletes, Exercise testing, Diagnostic accuracy, Cardiac remodeling, Echocardiography

## Abstract

**Background:**

Previous studies revealed a high rate of abnormal exercise test (ET) results in the absence of obstructive coronary artery disease (CAD) in asymptomatic athletes. The physiological background of this phenomenon is not well established. In particular, it is unclear whether sports-induced morphological cardiac adaptations are determinants of abnormal ET results. The main objective of this study was to investigate if healthy asymptomatic recreational and competitive athletes with abnormal ET results without obstructive CAD have a higher LV mass when compared with athletes with normal ET results.

**Methods:**

Seventy-three athletes with abnormal ET results without presence of obstructive CAD underwent echocardiographic assessment of LV mass, systolic and diastolic measurements. These data were compared with data from 73 athletes with normal ET results, matched for gender, age, body composition, sports characteristics and exercise capacity.

**Results:**

No significant increase in LV mass (161.9 ± 39 g vs. 166.9 ± 42.1 g, *p* = 0.461) was found between groups. Athletes with abnormal ET results had a significant thicker IVSd (9.7 ± 1.8 mm vs. 9.0 ± 1.7 mm, *p* = 0.014), higher IVSd/PWTd ratio (1.08 ± 0.20 vs. 1.00 ± 0.12, *p* = 0.011) and deceleration time (DT) was prolonged ((225.14 ± 55.08 vs. 199.96 ± 34.65, *p* = 0.003).

**Conclusion:**

Athletes with abnormal ET result did not show a higher in LV mass when compared to athletes with a normal ET result. However, a pattern of asymmetric cardiac remodeling, together with altered diastolic function is present. Due to small differences, cardiac remodeling only plays a limited role in the occurrence of positive ET results in athletes.

## Background

In order to obtain sports eligibility, athletes often undergo pre-participation screening (PPS) to identify genetic, congenital and/or acquired heart diseases in order to prevent the occurrence of sudden cardiac death (SCD) [1, 2]. In 1982, The first PPS protocol was introduced in Italy and effectively reduced cardiac events and SCD in young, active individuals [3]. Since then, several protocols have been established but many failed to identify those individuals with potentially lethal cardiac conditions [4]. Therefore, in addition to a general evaluation of the athlete’s overall health, a 12-lead resting electrocardiogram (ECG) was added in PPS protocols [5]. The ECG has proven to be an effective screening method as more than 80% of the individuals with underlying cardiac disorders, which account for the majority of SCD cases, had an abnormal ECG during PPS [6]. Because most exercise-related cardiovascular events occur during exericse, PPS also often includes exercise testing (ET), which is considered a low-cost non-invasive component for detection of myocardial ischemia in recreational and competitive athletes. However, several studies demonstrated a high prevalence of abnormal ET results, indicative for myocardial ischemia, in athletes in the absence of obstructive coronary artery disease (CAD [7–9]. Whereas this observation may be explained partly by a low a priori risk of CAD as compared to non-athletes, other sport-related explanations have also been proposed. First, exercise-induced ST-segment depression in athletes may be caused by microvascular dysfunction, inadequate density of myocardial capillaries relative to myocardial mass and compression of the microvascular arteries [10]. This theory is supported by a previous study [11] which showed that microvascular function and increase of capillary density is critically dependent on age. In older subjects, the increase in left ventricular mass outweighs the increase in capillary density and the microvascular function is altered in these subject when compared with younger subjects leading to a mismatch in oxygen demand and oxygen supply. Second, cardiac remodeling may play a role. In fact, different types of exercise training (endurance and strength) affect the cardiovascular system in different manners. This leads to various cardiac adaptations, including structural, functional and electrical changes, often referred to as “the athlete’s heart” [12]. Whereas electrical remodeling, including rest repolarization abnormalities, is known to induce false-positive ET results [13], the influence of morphological remodeling on ET results in athletes is not well established. In the general population, LV hypertrophy and hypertrophic cardiomyopathy were shown to be related to ST-segment depression independent from macrovascular CAD [10]. However, it remains unclear whether a sports-induced increase in LV mass, which is the most common morphological cardiac adaptation [12, 14], is a determinant of abnormal ET results.

Therefore, the main objective of this study is to investigate if healthy asymptomatic recreational and competitive athletes with abnormal ET results without obstructive CAD have a higher LV mass when compared with athletes with normal ET results.

## Methods

### Study design and population

This study was conducted as part of a single-center prospective study in asymptomatic recreational and competitive athletes who underwent PPS at Máxima Medical Center, Veldhoven, The Netherlands, between October 2014 and March 2017 and was approved by the local medical ethical committee. The main objective of the primary study was to investigate the oxygen uptake patterns during cardiopulmonary exercise testing in order to evaluate if microvascular dysfunction could play a role in the occurrence of abnormal ET results without obstructive CAD in athletes. In the primary study, athletes were defined as those who have practiced sports for at least 2.5 h a week for at least 30 weeks per year [15]. Athletes were excluded if they had complaints suggestive of cardiac disease (such as chest pain or impaired exercise tolerance). The exercise protocol that was used is extensively documented in the primary study [16]. An abnormal exercise test was defined as a minimum of 0.1 mV horizontal or downsloping ST-segment depression or elevation measured at least 80 or 60 ms after the J-point during exercise or recovery in three consecutive beats [13]. Athletes with abnormal ET results were referred to a cardiologist for further diagnostic evaluation including echocardiography, myocardial perfusion imaging and coronary angiography to rule out obstructive CAD. After written informed consent, subjects with abnormal ET result were included in the analysis. To evaluate if athletes with abnormal ET results without obstructive CAD have a higher LV mass when compared with athletes with normal ET results, athletes with abnormal ET results were matched 1:1 with athletes with normal ET results. Athletes who participated in the primary study and underwent echocardiography at Máxima Medical Center were selected for matching. The athletes with abnormal ET results were matched with athletes with normal ET results according to gender (1:1), age (+/− 3 years), body mass index (BMI) (+/− 0.5 kg/m^2^), body surface area (BSA) (+/− 0.2 m^2^), hours of sports per week (+/− 1 h), sport category (1:1) and maximal workload per kilogram (+/− 0.3 W/kg).

### Data collection

The following cardiovascular risk factors were collected through a questionnaire: hypertension, hypercholesterolemia, diabetes, use of tobacco and family history. This questionnaire also addressed the type of sports each athlete plays according to the Mitchell Classification of Sports [17] and the intensity of the sport (training hours per week). In addition, the following variables were obtained from the exercise test: resting mean arterial pressure (MAP), MAP at peak exercise, resting heart rate, maximal achieved heart rate, percentage of maximal predicted heart rate, maximal achieved workload and percentage of the predicted maximal workload.

### Resting electrocardiography

In all athletes, a 12-lead resting ECG was recorded in the supine position prior to the exercise test. The ECGs were analyzed following the international recommendations for the interpretation of ECGs in athletes [5]. The ECGs were classified as normal ECG findings or abnormal ECG findings that require further evaluation.

### Echocardiography

The athletes with a positive test result and who were referred to the department of cardiology received an echocardiogram including standard two-dimensional color, spectral Doppler measurements and additional measurements for cardiac remodeling to current standards [18]. Experienced senior echocardiography technicians, who were blinded for the initial abnormal ET result, performed the echocardiographic examination. Two individual cardiologists reported and validated the echocardiographic examination, both were not blinded for the initial abnormal ET result. For the calculation of the LV mass, the left ventricular end diastolic diameter (LVEDD), the end diastolic interventricular septal wall thickness (IVSd) and the end diastolic left ventricle posterior wall thickness (PWTd) were measured in millimeters from the parasternal long axis view. The following formula was used to calculate the LV mass in grams: 0.8 x (1.04 x (LVEDD + IVSd + PWTd)^3^ – (LVEDD)^3^) + 0.6. For the calculation of the relative wall thickness (RWT), the following formula was used: (IVSd + PWTd)/LVEDD [19]. To establish the cardiac diastolic function, the following echocardiographic parameters were measured: the maximal early ventricular filling velocity (E); the deceleration time (DT) of the early filling velocity; the maximal late ventricular filling velocity due to the atrial kick (A); the E/A ratio; the diastolic peak velocities of the mitral annulus measured at the interventricular septum and laterally, designated as E’ interventricular septum and E’ lateral, respectively; and the E/E’ ratio for both annulus measurements.

### Statistical analysis

Data were analyzed using SPSS 22 statistical software (SPSS Inc., Chicago, Illinois, USA). The distribution of all continuous variables was assessed using the Shapiro-Wilk test, and presented as mean ± SD or median (interquartile range). Between-group differences were assessed using one-way-analysis-of-variance to compare continuous parametric data and the Mann-Whitney U test for non-parametric data. Categorical data are presented as numbers and percentages and the chi-square test was used for between-group differences. A *p*-value of 0.05 was considered to be statistically significant.

## Results

**Fig. 1:**
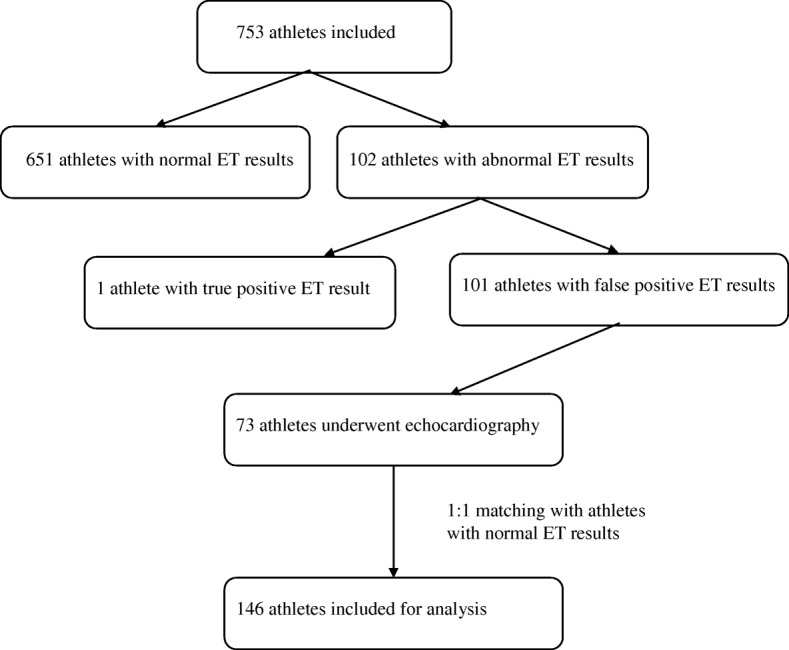
Flowchart of the study, Legend: ET, exercise testing

From a cohort of 753 athletes (mean age 45.7 ± 14.7 years, 81% male), 73 athletes (mean age 53.4 ± 9.6 years, 9.7%) with a positive ET result and who underwent echocardiography were selected (Fig. 1). All these athletes were referred to the department of cardiology for further diagnostic evaluation. Myocardial perfusion scintigraphy (MPS) was used in 57 athletes (78%), computed tomography coronary angiography (CCTA) was used in 15 athletes (21%) and direct coronary angiography was used in 1 athlete (1%). Diagnostic evaluation showed no abnormalities, suggestive for myocardial ischemia or significant coronary artery disease in 66 athletes (90%). Seven athletes (10%) showed a mean of 1.9 ± 1.1 ischemic segments of ischemia on MPS and subsequent CAG showed no obstructive CAD in all of these athletes. So, in all 73 athletes, no obstructive CAD was found. These 73 athletes were matched with athletes with normal ET results according to gender, age, BMI, BSA, hours of sports per week, sports category and maximal workload per kilogram as described above. Table 1 summarizes the characteristics of athletes with abnormal ET result who underwent echocardiography and of the matched control group with a negative exercise test. The study population consisted primarily of male athletes (86.3%), most frequently practicing high-static and high-dynamic sports (68.5%) of which cycling was the predominant practiced sport (*n* = 41, 82%). Soccer was the predominant practiced sport in the low-static/high-dynamic classified sports (*n* = 15, 71%).

**Table 1:** Comparison of the matched characteristics between athletes with abnormal ET results and the matched control group with normal ET results

	Abnormal ET results (*n* = 73)	Normal ET results (*n* = 73)	*P*-value
Male gender, n (%)	63 (86.3)	63 (86.3)	
Age (years)	53.4 ± 9.6	52.4 ± 9.5	0.507
BSA (m^2^)	1.97 ± 0.18	1.98 ± 0.16	0.705
BMI (kg.m^− 2^)	23.85 ± 2.6	24.63 ± 2.34	0.061
Hours sport/week	6.4 ± 3.5	6.7 ± 3.3	0.705
Sports classification, *n* (%)			
▪ HS/HD	50 (68.5)	50 (68.5)	
▪ LS/HD	21 (28.8)	21 (28.8)	
▪ MS/MD	2 (2.7)	2 (2.7)	
Workload per kilogram (W.kg^−1^)	4.32 ± 0.94	4.22 ± 0.92	0.530
ECG findings			0.343
● Normal	63 (86.3)	68 (93.2)	
● Abnormal	10 (13.7)	5 (6.8)	
Diagnostic evaluation, *n* (%)			
● MPS	57 (78.1)		
● CCTA	15 (20.5)		
● CAG	1 (1.4)		
Outcome diagnostic evaluation, *n* (%)			
● Normal	66 (90.4)		
Abnormal			
○MPS	7 (9.6)		
○Ischemic segments, mean	1.9 ± 1.1		
○Obstructive CAD, n	0		

*ET* exercise testing, *BSA* body surface area, *BMI* body mass index, *HS* high static, *HD* high dynamic, *LS* low static, *HD* high dynamic, *MS* mid static, *MD* mid dynamic, *W* workload, *MPS* myocardial perfusion scintigraphy, *CT* computed tomography coronary angiography, *CAG* coronary angiography, *CAD* coronary artery disease

The comparison of the exercise parameters between athletes with abnormal ET results and the athletes with normal ET results are demonstrated in Table 2. No significant differences were observed in MAP at peak exercise, heart frequency at rest, maximal achieved workload and percentage of the predicted maximal workload. The maximal heart rate (174.8 ± 16.5 bpm vs. 167.8 ± 14.5 bpm, *p* = 0.007) and the percentage of the predicted maximal heart rate (99.7 ± 8.0 versus 95.4 ± 7.7, *p* = 0.001) were significantly higher in the group with the abnormal ET results. The MAP at rest was also lower in the group with the abnormal ET results (97.2 ± 10.4 vs. 102.1 ± 11.9 mmHg).

**Table 2:** Comparison of exercise parameters between athletes with abnormal and normal ET results

	Abnormal ET results (*n* = 73)	Normal ET results (*n* = 73)	*P*-value
Rest MAP (mmHg)	97.2 ± 10.4	102.1 ± 11.9	0.009*
Max MAP (mmHg)	119.5 ± 12.3	121.6 ± 11.9	0.301
Rest HF (bpm)	66.3 ± 13.9	67.8 ± 13.6	0.516
Max HF (bpm)	174.8 ± 16.5	167.8 ± 14.5	0.007*
% pred HF max	99.7 ± 8.0	95.4 ± 7.7	0.001*
Wmax (watt)	331.8 ± 73.2	331.9 ± 72.2	0.994
% pred Wmax	152.6 ± 22.6	153.8 ± 26.2	0.776

^*^P < 0.05

*ET* exercise testing, *Rest* variables measured in rest, *Max* variables measured at maximal exercise, *MAP* mean arterial pressure, *HF* heart frequency, *% pred HF max* percentage predicted maximal heart frequency, *Wmax* maximal workload, *% pred Wmax*, percentage predicted maximal workload

Table 3 shows the echocardiographic findings in athletes with abnormal ET results and the athletes with normal ET results. Athletes with abnormal ET results showed no significant increased LV mass when compared with athletes with normal ET results (161.9 ± 39 g vs. 166.9 ± 42.1 g, *p* = 0.461). When corrected for the BSA, also no significant difference was observed between the two groups (82.1 ± 18 g.m-2 vs. 84.2 ± 19.9 g.m-2, *p* = 0.501). The LVEDD was significantly lower in athletes with abnormal ET result (48.4 ± 4.4 mm vs. 51.0 ± 5.3 mm, *p* = 0.002). The end diastolic IVS was significantly thicker in the group of athletes with abnormal ET result (9.7 ± 1.8 mm vs. 9.0 ± 1.7 mm, *p* = 0.014). No significant difference was observed in the end diastolic PWT between the two groups (*p* = 0.718). There was a significantly higher IVSd/PWTd ratio (1.08 ± 0.20 vs. 1.00 ± 0.12, *p* = 0.011) and RWT (0.38 ± 0.06 vs. 0.36 ± 0.06, *p* = 0.044) in athletes with abnormal ET result. Regarding indicators of LV diastolic function, there was no significant difference in early ventricular filling velocity (E), late ventricular filling velocity (A) and the E/A ratio between the two groups (*p* = 0.281, *p* = 0.762, *p* = 0.363, respectively). Yet, the deceleration time of the early filling velocity was significantly prolonged in athletes with abnormal ET results (225.14 ± 55.08 vs. 199.96 ± 34.65, *p* = 0.003). No significant difference was found in the E/E’ septal and lateral ratio between the two groups (*p* = 0.904, *p* = 0.936, respectively).

**Table 3:** Echocardiographic findings in athletes with abnormal and normal ET results

	Abnormal ET results (*n* = 73)	Normal ET results (*n* = 73)	*P*-value
LV mass (g)	161.9 ± 39.0	166.9 ± 42.1	0.461
LV mass/BSA (g.m^− 2^)	82.1 ± 18.0	84.2 ± 19.9	0.501
LVEDD (mm)	48.4 ± 4.4	51.0 ± 5.3	0.002*
IVSd (mm)	9.7 ± 1.8	9.0 ± 1.7	0.014*
PWTd (mm)	9.1 ± 1.4	9.0 ± 1.4	0.718
LVESD (mm)	32.3 ± 2.7	34.1 ± 4.4	0.005*
ESWS (g.cm^− 2^)	66.7 ± 16.3	81.8 ± 26.3	< 0.001*
IVSd/PWTd ratio	1.08 ± 0.20	1.00 ± 0.12	0.011*
RWT	0.38 ± 0.06	0.36 ± 0.06	0.044*
MV max flow E (m.s^− 1^)	0.694 ± 0.160	0.724 ± 0.160	0.281
MV DT E (ms)	225.14 ± 55.08	199.96 ± 34.65	0.003*
MV max flow A (m.s^− 1^)	0.591 ± 0.143	0.584 ± 0.111	0.762
E/A ratio	1.22 ± 0.33	1.28 ± 0.36	0.363
E/E’ septal ratio	7.73 ± 1.91	7.69 ± 1.62	0.904
E/E’ lateral ratio	5.68 ± 1.58	5.77 ± 1.64	0.936

^*^P < 0.05

*ET* exercise testing, *LV* left ventricular, *LVEDD* left ventricular end diastolic diameter, *IVSd* interventricular septal wall thickness at diastole, *PWTd* posterior wall thickness at diastole, *RWT* relative wall thickness, *MV* max flow E, maximal early ventricular filling velocity, *MV DT E* deceleration time of the early filling velocity, *MV max flow A* maximal late ventricular filling velocity, *E’ septal* diastolic peak velocity of the mitral annulus measured at the interventricular septum, *E’ lateral* diastolic peak velocity of the mitral annulus measured lateral

## Discussion

This study showed that athletes with a positive ET result in the absence of obstructive CAD do not have a higher LV mass than athletes with normal ET results. These athletes, however, did show a smaller LVEDD, a higher RWT and asymmetric remodeling due to an increased IVS thickness, as well as a prolongation of the DT of the early filling velocity of the LV suggestive of an altered diastolic function. It should be acknowledged that these differences were small and values were still within the normal range. Therefore, these results suggest that sport-induced morphological adaptations only play a limited role in the development of STT changes during exercise.

To the authors’ knowledge, this is the first prospective study evaluating the relationship between LV remodeling and ET results in healthy asymptomatic athletes. In the present study, there was no significant difference in absolute LV mass and LV mass indexed to BSA between the athletes with abnormal ET results and the athletes with normal ET results. These findings are in line with previous studies [20, 21] that include smaller study populations. Despite the fact that the present study did not show a relationship between LV mass and abnormal ET results, the RWT was significantly higher in athletes with abnormal ET results than in athletes with a normal ET result (0.38 ± 0.06 versus 0.36 ± 0.06, *p* = 0.044). The present study also showed a significantly decreased LVEDD and increased IVS thickness at the end diastole in athletes with abnormal ET results, leading to a significantly higher IVS/PWT ratio. These findings suggest that athletes with abnormal ET result show a pattern of asymmetric cardiac remodeling.

The two groups of athletes in the present study showed no significant difference in the delivered maximal workload. However, a significantly higher maximum heart rate was observed in athletes with abnormal ET results. According to the Fick principle (V̇_O2, max_ = cardiac output x [arterial - venous O_2_ difference]), one can assume that there is a reduced stroke volume (SV) in athletes with abnormal ET result, as there is a linear relationship between the workload and V̇_O2_ [22]. A possible explanation of the reduced SV could be the presence of a smaller left ventricular cavity expressed as a reduction in the LVEDD due to the asymmetric remodeling seen in the present study. The findings of a significant higher achieved maximum heart rate reflect necessary physiological adaptations to the observed cardiac remodeling in order to obtain the necessary increase in cardiac output.

The asymmetric remodeling pattern may be explained by Poiseuille’s law (pressure = flow x resistance). During exercise, and especially in dynamic sports activities, cardiac output (flow) increases more than total peripheral resistance (TPR), resulting in an increase in LV afterload, which initiates cardiac remodeling. According to the law of Laplace, an increase in afterload causes an increase in wall stress. In order to reduce wall stress, concentric remodeling occurs with an increase in overall wall thickness. However, in some patients, asymmetric remodeling develops rather than concentric remodeling. In particular, asymmetric remodeling is commonly observed in patients with an increase in LV afterload due to aortic stenosis and systemic hypertension (10–25%) [23]. This may be explained by the fact that the ventricular septum has a larger bending radius than the posterior wall. During contraction, this larger bending radius leads to greater myocardial stress of the septal wall. This initiates a more pronounced hypertrophic response leading to asymmetric remodeling of the septal wall [24]. The same mechanism may be responsible for the findings in athletes in the present study. Yet, it remains unclear why only some of the athletes in this study express asymmetric remodeling. One could speculate that these athletes have a genetic predisposition for developing an increase in myocardial wall thickness in response to an increase in LV afterload [24]. It is also known that an increase in afterload leads to an impairment of myocardial relaxation [25]. This theory is supported by Hayashida et al. [26], who show that a greater impairment of myocardial relaxation is observed with increasing wall thickness. In this way, afterload and (asymmetric) cardiac remodeling can alter diastolic LV function and eventually lead to diastolic dysfunction.

This altered diastolic function could possibly be an explanation for the observed ST-segment depression in athletes without the presence of epicardial CAD. As shown in the present study, there was a significant prolongation of the DT of the early left ventricular filling velocity in athletes with abnormal ET results. This prolonged DT reflects an impaired left ventricular relaxation. Ventricular relaxation is one of the major determinants of diastolic filling and is characterized by the duration of the decrease of LV pressure after systole. A prolonged DT indicates that LV end diastolic pressure (LVEDP) is elevated. It is known that a minimal perfusion pressure is necessary to maintain patency of the coronary microvasculature [27]. As a result of an elevated LVEDP, the critical closing pressure of the coronary microvasculature could be exceeded. Due to this phenomenon, the perfusion gradient is compromised, resulting in a reduced coronary flow. In turn, the reduced coronary flow leads to an ischemic response, which further aggravates the LVEDP through ischemia-induced alterations in the diastolic pressure–volume relationship.

Another possible explanation for the ST-segment depression could also be related to the observed cardiac remodeling. It is known that an increase in myocardial mass should be evenly compensated with an increase in angiogenesis to fulfill the requirements for increased oxygen consumption [11]. However, this response has been found to be critically dependent on age [11], as angiogenesis does not outweigh the increase in LV mass in the elderly [28]. The present study included athletes with a mean age of 52.9 years. It is feasible to think that there might be an imbalance between myocardial mass and angiogenesis, especially in the group of athletes with asymmetric cardiac remodeling.

It has also been shown that [27, 29] this imbalance leads to a reduced vasodilator reserve. A previous study [11] shows that an active vasomotor tone is required to maintain subendocardial perfusion by creating a flow gradient to the deeper myocardial layers. In the case of a defect in the vasomotor tone, there is an impaired regulation of myocardial perfusion due to the diminished gradient from epicardium to endocardium. This dysfunctional vasodilation may be caused by a reduction in availability of the endothelium-derived relaxing factor (EDRF), also known as nitric oxide (NO), and an increase of endothelin-1 (ET-1) levels. Due to the imbalance between vasodilation and vasoconstriction, an endothelial vasodilator dysfunction can develop [30]. Therefore, endothelial dysfunction could be an explanation of exercise-induced ischemia in athletes, although previous studies show conflicting results of both increased endothelial function [31], similar [32] and reduced endothelial function [14] in athletes. In athletes, it is known that an increased myocardial blood transition time enables higher oxygen extraction levels together with a lower myocardial blood flow and higher vascular resistance [33]. All these adaptations are key to deliver adequate oxygen supply to the myocardium in response to the needs during exercise in the athletic heart. In the case of an imbalance between myocardial mass and angiogenesis together with a reduced vasomotor tone, myocardial oxygen delivery is reduced, which can induce exercise-induced ischemia.

Myocardial ischemia with ST-segment depression can also occur due to cardiac compressive forces. During basal conditions, systolic contraction impedes myocardial blood flow resulting in primarily diastolic filling of the coronary vasculature. However, during exercise 40–50% of the coronary blood flow occurs during systole as the diastolic interval decreases with higher heart rates. It is feasible to think that the observed increase in septal wall thickness, together with the above-mentioned imbalance, compresses the coronary microvasculature. These factors, solely or combined, could cause the observed ST-segment depression as an expression of myocardial ischemia.

### Clinical implications

The high prevalence of abnormal ET results in athletes requires consideration, because an erroneous diagnosis may have important consequences. For instance, a false diagnosis of cardiac disease warrants unnecessary activity restriction or even disqualification from sports, while an incorrect diagnosis may jeopardize the life of an athlete [34]. In addition, athletes with abnormal ET results undergo invasive diagnostic procedures, which are accompanied with higher health care costs. In order to reduce the high number of abnormal ET results, it is necessary to elucidate associated mechanisms and identify predictors that can be used to improve the positive predictive value of electrocardiography in asymptomatic athletes. This study shows a possible relationship between LV asymmetric remodeling and the occurrence of abnormal ET results. However, given the small differences in echocardiographic values between groups, cardiac remodeling only seems to play a limited role. Therefore, other sports-related mechanisms that may be related to the development of STT changes during exercise, such as microvascular dysfunction, insufficient density of myocardial capillaries relative to myocardial mass and compression of the microvascular arteries due to high ventricular filling pressure, should be explored.

### Limitations

There are several limitations that should be acknowledged. First, LV mass was measured via echocardiography. M-mode echocardiography is the most widely used technique in the evaluation of the LV mass, because of the relatively low costs and the rapid and non-invasive character. However, M-mode echocardiography has important limitations particularly in subjects with abnormal LV geometry as it is known that the accuracy of LV mass measurement declines in dilated left ventricles [35]. Also, it is known that M-mode echocardiography overestimates LV mass when compared with magnetic Resonance Imaging (MRI) and there is a high test-retest variability in LV mass among one single individual of approximately 35 g (the smallest detectable change with 95% confidence interval) [36]. Therefore, in order to detect a change in LV mass of at least 10 g, approximately 200 subjects per group would be necessary [37]. Another limiting factor is the intra- and interobserver variability in the measurement of LV mass. Literature showed that there is an intraobserver variability of 19 g and an interobserver variability of 24 g [38]. MRI is the gold standard for accurate assessment of the LV mass, but this technique is relatively expensive, time consuming and not feasible for all patients [39]. Therefore, it is less suitable for routine clinical screening for cardiac remodeling in athletes. Second, the primary study was not powered to detect a difference in LV mass in athletes which could lead to a type II error in the present study. Finally, the study population consisted primarily of male athletes who practice high-static, high-dynamic sports. Therefore, it was not possible to make a reliable statement about the correlation of LV remodeling on abnormal ET results in female athletes and athletes who perform low-static, low-dynamic exercise or other types of sports.

## Conclusion

Athletes with abnormal ET result did not show a higher LV mass when compared to athletes with a normal ET result. However, these athletes did show a pattern of asymmetric cardiac remodeling due to a greater IVS thickness, together with an altered diastolic function as indicated by a prolonged DT. Yet, as these differences were small and values were still within the normal range, these results suggest that cardiac remodeling only plays a limited role in the occurrence of abnormal ET results in athletes.

## Abbreviations

PPS: Pre-participation screening ET Exercise testing CAD Coronary artery disease LV Left ventricular/left ventricle ECG Electrocardiography LVEDD Left ventricular end diastolic dimension IVSd Interventricular septal thickness end diastole LVPWd Left ventricular posterior wall thickness end diastole RWT Relative wall thickness DT Deceleration time
